# Bioassay-Guided Assessment of Antioxidative, Anti-Inflammatory and Antimicrobial Activities of Extracts from Medicinal Plants via High-Performance Thin-Layer Chromatography

**DOI:** 10.3390/molecules28217346

**Published:** 2023-10-30

**Authors:** Marko D. Jović, Snezana Agatonovic-Kustrin, Petar M. Ristivojević, Jelena Đ. Trifković, David W. Morton

**Affiliations:** 1 Innovation Centre of the Faculty of Chemistry Ltd., University of Belgrade, Studentski Trg 12-16, 11158 Belgrade, Serbia; markojovic@chem.bg.ac.rs; 2 Department of Pharmaceutical and Toxicological Chemistry Named after Arzamastsev of the Institute of Pharmacy, I.M. Sechenov First Moscow State Medical University (Sechenov University), 119991 Moscow, Russia; d.morton@latrobe.edu.au; 3 Department of Rural Clinical Sciences, La Trobe University, Edwards Road, Bendigo, VIC 3550, Australia; 4 Department of Analytical Chemistry, Centre of Excellence for Molecular Food Sciences, University of Belgrade, Studentski Trg 12-16, 11158 Belgrade, Serbia; jvelicko@chem.bg.ac.rs

**Keywords:** antibacterial, anti-inflammatory, antioxidants, bioautography, effect-directed analysis, Fourier-transform infrared spectroscopy, high-performance thin-layer chromatography

## Abstract

Natural products and their analogues have contributed significantly to treatment options, especially for anti-inflammatory and infectious diseases. Thus, the primary objective of this work was to compare the bioactivity profiles of selected medicinal plants that are historically used in folk medicine to treat inflammation and infections in the body. Chemical HPTLC fingerprinting was used to assess antioxidant, phenolic and flavonoid content, while bioassay-guided HPTLC was used to detect compounds with the highest antibacterial and anti-inflammatory activities. The results of this study showed that green tea leaf, walnut leaf, St. John’s wort herb, wild thyme herb, European goldenrod herb, chamomile flower, and immortelle flower extracts were strong radical scavengers. Green tea and nettle extracts were the most active extracts against *E. coli*, while calendula flower extract showed significant potency against *S. aureus*. Furthermore, green tea, greater celandine, and fumitory extracts exhibited pronounced potential in suppressing COX-1 activity. The bioactive compounds from the green tea extract, as the most bioactive, were isolated by preparative thin-layer chromatography and characterized with their FTIR spectra. Although earlier studies have related green tea’s anti-inflammatory properties to the presence of catechins, particularly epigallocatechin-3-gallate, the FTIR spectrum of the compound from the most intense bioactive zone showed the strongest anti-inflammatory activity can be attributed to amino acids and heterocyclic compounds. As expected, antibacterial activity in extracts was related to fatty acids and monoglycerides.

## 1. Introduction

Despite the advances in medical research and modern treatment methodologies with a plethora of synthetic pharmaceuticals, the importance of medicinal plants as a source of pharmaceutical products remains the same. The products derived from medicinal plants are often among the primary remedies sought for alleviating health-related issues as they are readily accessible, generally recognized as safe for use with minimal adverse effects, and well-tolerated [1,2]. About 80% of 122 plant-derived drugs are linked with their original traditional uses. The therapeutic properties of medicinal plants are attributed to their secondary metabolites, which they produce as a defence mechanism against pathogens, predators, and UV/Vis radiation [3]. These compounds possess a variety of health promoting properties, such as antimicrobial, antioxidative, anticancer, and anti-inflammatory activities [4]. 

Given that plant extracts are very complex component matrices containing hundreds of compounds, planar chromatography hyphenated with biological detection, termed bioautography, has been established in recent years as an ideal screening method [5]. Bioautography, as a means of highly target-directed detection of bioactive molecules, involves chromatographic separation of compounds followed by a dipping or spraying of chromatograms in a free radical or enzymatic solution, as well as in a microbial suspension. Planar chromatography, in the form of thin-layer chromatography (TLC), or in its most advanced form, high-performance thin-layer chromatography (HPTLC), provides a robust separation technique. The open layer of the stationary phase allows solvent evaporation and makes it possible to integrate biological detection directly onto a stationary phase, making it suitable for effect-directed analysis [6]. Due to its simplicity, time efficiency, and minimal equipment requirements, bioautography has been recognized as a tool for antimicrobial and enzyme inhibitor detection. After target detection of bioactive compounds, HPTLC has been successfully coupled with additional structure elucidation methods, such as absorption or fluorescence spectroscopy, attenuated total reflectance-Fourier transform infrared (ATR-FTIR) spectroscopy, mass spectrometry (MS) and nuclear magnetic resonance (NMR) [7,8]. 

The present study was designed with the aim to compare the phytochemical and bioactivity profiles of ethanol-based extracts from 19 medicinal plants via HPTLC chemical reagent derivatization and HPTLC bioautography. All selected plants belong to the most represented botanical families of medicinal flora (i.e., Lamiaceae, Apiaceae, Asteraceae and Rosaceae families) [9]. They are recognized for their use against bacterial, fungal, and viral infections [10,11] and are traditionally used in folk medicine to reduce inflammations and infections in the body. HPTLC bioautography was used for screening and detection of antioxidant, anti-inflammatory and antimicrobial compounds, with the most bioactive compounds characterised via ATR-FTIR spectroscopy. Free radical, enzymatic and bacterial in situ assays were used to assess antioxidant activity, COX-1 enzyme inhibition and antibacterial activity. Combination of HPTLC separation with in situ bioassays, followed by spectrophotometric characterization of bioactive compounds, enables detection of bioactive compounds in complex samples, such as plant extracts, without the need for extraction. Running an in vitro assay on a whole crude extract without previous separation is not very informative, as plant extracts present extremely complex, multicomponent mixtures, usually composed of hundreds to thousands of secondary metabolites that provide mixed effects or in some cases have opposite effects that cancel each other out. Therefore, a combination of HPTLC with bioassay-guided detection of bioactive zones in chromatograms enabled the selection of only compounds with targeted biological activities for further characterization.

## 2. Results and Discussion

### 2.1. HPTLC Fingerprints and Chemical Profiling

**Figure 1:**
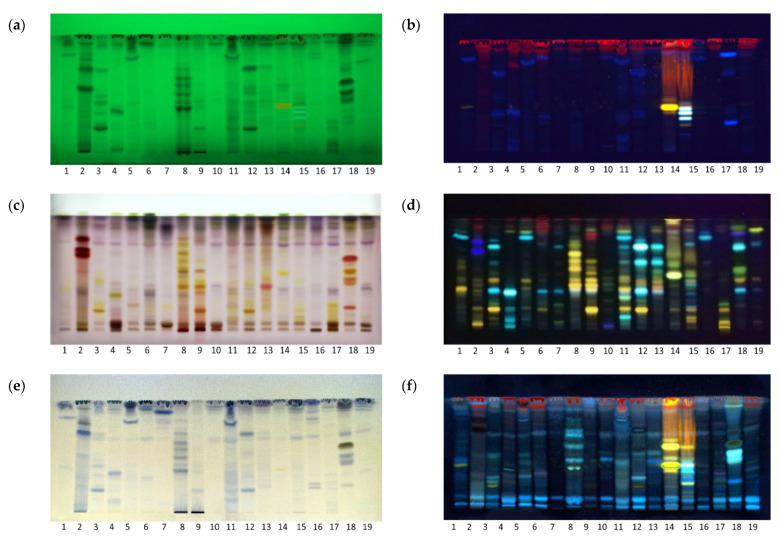
HPTLC fingerprints of 19 herbal extracts visualized under (**a**) UV 254, (**b**) UV 366 nm, (**c**) with vanillin sulfuric acid (VSA) reagent, white light (**d**) natural product natural product/polyethylene glycol (NP/PEG) reagent, UV 366 nm (**e**), ferric chloride reagent, white light (**f**) aluminium chloride, UV 366 nm. Mobile phase, ethyl acetate: toluene: formic acid: water (16:4:3:2 *v*/*v*/*v*/*v*).

Unique chromatographic fingerprints of extracts were obtained using a mobile phase of ethyl acetate: toluene: formic acid: water (16:4:3:2, *v*/*v*/*v*/*v*) optimized for the maximal separation of phenolics. The separated compounds were visualized by their distinct colors, fluorescence under UV light, or after derivatization (Figure 1).

Aromatic compounds and conjugated systems were seen as dark zones against a green background under 254 nm as they absorb UV light and quench the green fluorescence of the plate fluorescent indicator (Figure 1a), while red-, yellow- and blue-colored fluorescent zones were seen under UV 366 nm (Figure 1b), as the fluorescence of some substances was excited by long-wave UV light. For those compounds which were not visible under UV light, plates were exposed to chemical reagents. The vanillin–sulfuric acid (VSA) reagent was used as a reagent for higher alcohols, phenols, steroids and terpenoids [12]. VSA produced a range of colors, depending on the types of compounds, although the color and intensity of the observed zone also depends on the concentration of the compound separated on the plate. Proanthocyanidins (oligomeric flavonoids) are seen as bright pink [13], monoterpenes and steroids as bright yellow to violet zones, while flavonoids are colored yellow [14]. Thus, red and pink colors suggest the presence of phenolic derivatives and a rich content of proanthocyanidins in green tea, walnut, St. John’s wort and immortelle (Figure 1c, Tracks 2, 8, 9 and 18), while violet color suggest the presence of various terpenoid derivatives at the upper parts of most chromatograms (*R*_F_ = 0.8–0.9).

Two different detection methods were used for the visualization of flavonoids: (a) spraying with NP/PEG solution (Figure 1d), and (b) spraying with AlCl_3_ reagent. These were both evaluated under UV 366 nm (Figure 1f). The NP/PEG reagent is an analytical reagent used for the detection of flavonoids, isoflavonoids and phenolic acids, by enhancing their natural fluorescence and forming different colored zones under UV light depending on the structure of the molecule. Molecules with one hydroxyl group appear yellow, molecules with two hydroxyl groups appear orange yellow, and molecules with three hydroxyl groups appear green [15]. Since a limited number of flavonoids exhibit native fluorescence, non-fluorescent flavonoids are detected as fluorescent complexes after complexation with highly fluorescent chelate Al^3+^ ions [16]. After spraying with AlCl_3_, flavonoids were visualized as light green fluorescent zones under UV 366 nm [17]. The walnut extract (Figure 1f, Track 8) emerged as the extract with the highest content of flavonoids due to the presence of multiple fluorescent zones.

Ferric chloride is a highly specific spray reagent, used mainly to visualize phenols, although some carbonyl compounds with stable ketone enolates may also be visualized [18]. Derivatization with a neutralized FeCl_3_ solution produced blue zones against a pale yellow background that corresponds to colored complexes of phenolic compounds with Fe^3+^ ions (Figure 1e). According to the number and intensity of the zones by visual inspection of the HPTLC chromatograms, extracts of green tea (Track 2), walnut (Track 8), wild thyme (Track 11) and immortelle (Track 18) emerged as those with the highest phenolic content (Figure 1e).

### 2.2. HPTLC Bioassay Profiling 

**Figure 2:**
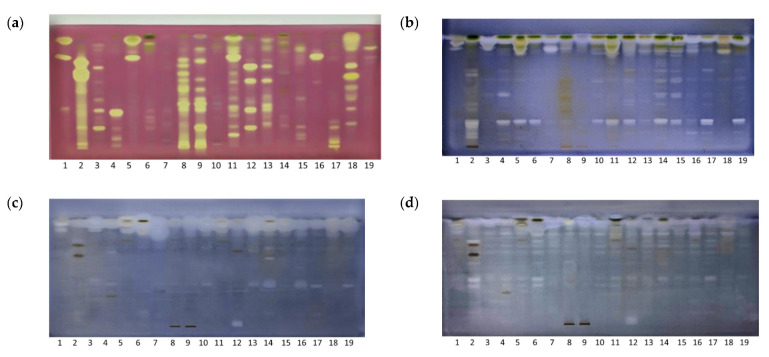
HPTLC fingerprinting of herbal extracts under white light after (**a**) DPPH• scavenging assay; (**b**) COX-1 inhibitory assay; (**c**) MTT antimicrobial assay against *S. aureus*; and (**d**) MTT antimicrobial assay against *E. coli*. Mobile phase, ethyl acetate: toluene: formic acid: water (16:4:3:2 *v*/*v*/*v*/*v*).

Bioactive zones on chromatograms were detected by coupling HPTLC with bioassays. A DPPH• scavenging assay was used to evaluate antioxidant activity, an in situ enzymatic bioassay was used to screen for COX-1 inhibitors, while microbial detection using the MTT colorimetric bioassay was used to assess antibacterial activity against *E. coli* and against *S. aureus* in the extracts (Figure 2a–d).

Extract antioxidant activity was evaluated using both the colorimetric and the HPTLC-based DPPH• assays (Figure 2a). On the plate, antioxidants appear as yellow zones against a purple background, whereas in the spectrophotometric approach, activity is monitored through the reduction in violet color intensity. The advantage of HPTLC over the spectrophotometric method is that it enables detection of the individual components contributing to the antioxidant potential. However, interactions among the separated compounds and functional groups on the stationary phase can influence the manifestation of their activity [19].

Multiple zones of antioxidants across all samples were detected with the HPTLC-DPPH• scavenging assay. These samples can be categorised into three groups based on their antioxidant activity: extracts of green tea (Track 2), walnut (Track 8), St. John’s wort (Track 9), wild thyme (Track 11), European goldenrod (Track 12), chamomile (Track 13), and immortelle (Track 18) showed strong antioxidant activity; extracts of calendula (Track 7), grater celandine (Track 14), fumitory (Track 15), and dandelion (Track 19) showed weak activity; and the remaining extracts showed moderate antioxidant activity. Notably, bands with *R*_F_ ≈ 0.2, 0.4, 0.65, 0.75, and 0.95 contributed significantly to the antioxidant activity. The findings on the chromatogram were corroborated by the spectrophotometric tests. The colorimetric method affirms the highest DPPH• activity in green tea extracts, followed by walnuts, St. John’s wort, and immortelle (Table 1). Nonetheless, it is essential to acknowledge that other compounds such as terpenoids, vitamins, amino acids, and others, collectively influence the overall observed antioxidant activity [20]. According to the literature, the phenolic compounds found in green tea are rich in catechins. Epigallocatechin-3-gallate (EGCG) is one of the most abundant and has been identified as having the highest antioxidant potential compared to the others [21]. Moreover, amino acids which play an important role in the chemical composition of green tea, and thus in the taste of tea, also exhibit significant antioxidative properties. L-theanine, the primary amino acid found in green tea, is structurally related to glutamate, and is a precursor of the main endogenous non-enzymatic antioxidant glutathione. The strong antioxidant activity of L-theanine is linked with the regulation of antioxidant-related enzyme activity [22].

The anti-inflammatory properties of the plant extracts were assessed using a COX-1 inhibitory assay. COX-1 and COX-2 enzymes catalyze the oxidation of arachidonic acid to prostaglandin (PG)G2, which is subsequently reduced to PGH2 through peroxidase activity. In the HPTLC assay presented here, arachidonic acid was used as a substrate, while the visualization of COX inhibitors occurred through the oxidation of Wurster’s reagent, acting as a peroxidase co-substrate. This chemical reaction led to the formation of a blue-colored plate background against the white active zones of inhibition on the chromatogram. Chromatogram analysis revealed active regions at *R*_F_ ≈ 0.25 across the majority of the examined extracts (Figure 2b). Based on the quantity and intensity of white zones, certain plants like green tea, greater celandine, and fumitory exhibited pronounced potential in suppressing COX-1 activity. Conversely, walnut leaf and St. John’s wort displayed fewer inhibitory zones, indicative of comparatively weaker anti-inflammatory effects under the given conditions. Comparative assessment with other chromatograms allowed for presumptions about the class of compounds demonstrating inhibitory activity. For instance, intensely blue-colored zones corresponding to flavonoids emerged in green tea extract at *R*_F_ ≈ 0.1 and 0.25 and in fumitory at *R*_F_ ≈ 0.25, subsequent to derivatization with an AlCl_3_ solution (Figure 1f). These flavonoid-associated zones exhibited COX-1 inhibitory activity. Flavonoids are recognized for their anti-inflammatory attributes, particularly when combined, exhibiting significant synergistic effects in reducing the release of primary proinflammatory mediators [23].

Antibacterial activity against the test organisms, *S. aureus* and *E. coli,* was assessed with a colorimetric MTT assay. Treatment of infections caused by these strains represents a growing challenge due to the escalation of resistance towards conventional antibiotics. *S. aureus* is a prominent hospital and community-associated pathogen, responsible for numerous infections, from mild skin and soft tissue infections to infective endocarditis, osteomyelitis, bacteremia, and life-threatening pneumonia. It is notably predisposed to developing antibiotic resistance, perhaps the most known variant being methicillin-resistant *S. aureus* (MRSA) [24]. *E. coli* is the most common Gram-negative bacterium accountable for an array of diseases, as an outcome of both community and hospital acquired clinically bloodstream infections, significantly contributing to mortality across all age groups [25]. 

The applied assay is based on the activity of oxidoreductases present in viable bacterial cells which reduces yellow-colored MTT to purple formazan. The prepared plate was immersed in a bacterial suspension, and subsequently incubated to facilitate bacterial growth on the plate. Regions on the plate corresponding to the antibacterial compounds of the extracts manifest as faint yellow zones against a blue–purple background. The obtained chromatograms exhibit a greater number of active zones against *E. coli* in comparison to *S. aureus*. Intense regions observed at the forefront of the mobile phase signify the strong antibacterial activity of lipophilic components within the extracts, which were not effectively separated under the applied chromatographic conditions. According to the intensity of the antibacterial zones, green tea and nettle are the most active extracts against *E. coli*, while for *S. aureus*, the calendula flower demonstrated significant potency attributed to the active region at *R*_F_ ≈ 0.9, followed by the greater celandine herb. This was due to the higher number of active zones in comparison to other extracts (Figure 2c,d, Tracks 2, 6).

### 2.3. Quantification of Phenolics and Biological Activity of Medicinal Plants

HPTLC chromatograms corresponding to phenolic profile, antioxidant, COX-1 inhibitory, and antibacterial activity were used for quantification of total phenolics content (TPC) and different biological activities of examined medicinal plants. Peak areas of target bioactivity zones were processed with VideoScan^®^ software (version 1.02, CAMAG, Muttenz, Switzerland) and the TPC/biological activity per extract was compared with the activity of a reference standard. TPC and antioxidant activity were expressed as milligrams of gallic acid per gram of dry extract, COX-1 inhibitory activity as milligrams of salicylic acid equivalent (SAE) per gram of extract, and antibacterial activity as milligrams of streptomycin equivalent (StrpE) per gram of extract. Standard curves for gallic acid, salicylic acid, and streptomycin were constructed by plotting the applied amounts (µg) against corresponding areas measured in pixels. Statistical parameters for method validation (i.e., linearity range, precision and sensitivity) are presented in Supplementary tables (Tables S1–S3). 

Based on the FeCl_3_ test, the extracts of green tea, walnut, and immortelle, exhibited the highest phenolics content (Table 1). In contrast, extracts of St. John’s wort and wild thyme, together with green tea, walnut, and immortelle, demonstrated the highest free radical scavenging activity. We note that the DPPH radical values were higher compared to those obtained with FeCl_3_. This difference can be attributed to the antioxidant activity of other compound classes present in the extracts, as both tests utilized gallic acid equivalents for activity expression.

Nettle and green tea extract were identified as the most promising antimicrobial agents compared to the rest of investigated herbs, because nettle exhibited the strongest activity against *E. coli*, while green tea extract displayed the highest activity against *E. coli* and was the most potent COX-1 inhibitor (Table 1). Literature supports the robust antibacterial activity of nettle due to its rich phenolics content as well as presence of other naturally active compounds such as tannins, terpenoids, and alkaloids [26]. Conversely, St. John’s wort and immortelle, although rich in phenolic compounds and effective against the DPPH radical, proved less potent in bioassays (Table 1). 

To further evaluate the potential of investigated medicinal herbs, spectrophotometric determination of total phenolic content (TPC), total flavonoid content (TFC) and radical scavenging activity (RSA) were performed, as more often used methods in relation to the HPTLC approach. While colorimetric methods may lack the capability to reveal details about the specific constituents within an extract, this approach provides results that remain uninfluenced by the interactions between the functional groups on the stationary phase and those present in the compounds of interest. Comparing the results derived from colorimetric and HPTLC methods can offer a more objective assessment of the content of active compounds in the examined extracts. Statistical parameters for calibration are given in Table S3. 

Both the HPTLC method and the spectrophotometric tests show the highest values for phenolic content and antioxidant activity for green tea extract, while walnut, St. John’s wort and immortelle extracts also have high TPC. Walnut extract has the highest TFC, a result that closely aligns with the findings obtained using the HPTLC method following derivatization with AlCl_3_ solution (Table 1).

### 2.4. Target Analysis of Most Promising Extracts

**Figure 3:**
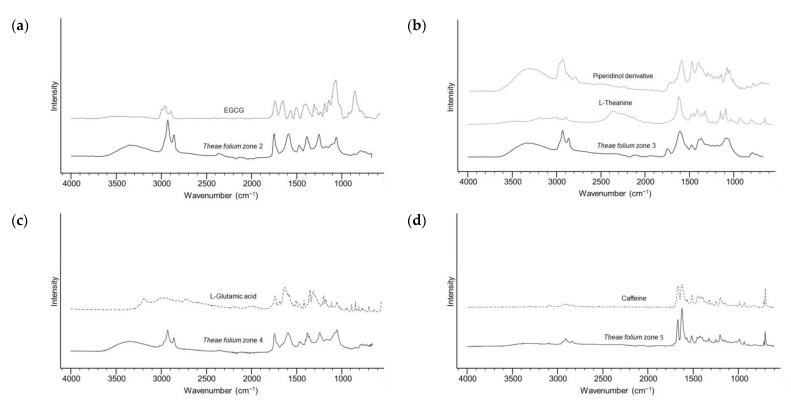

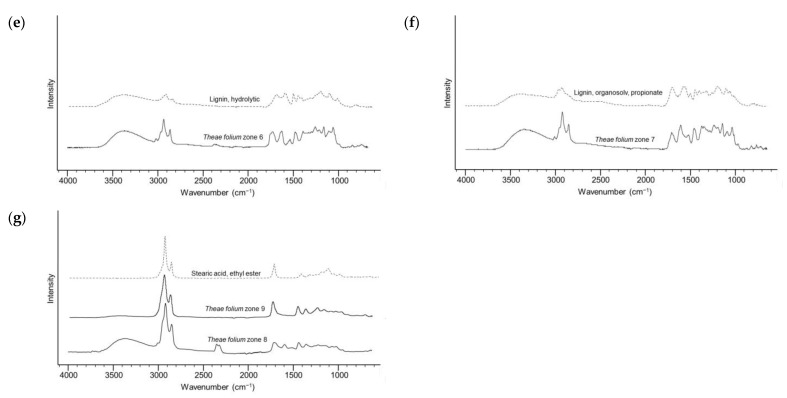
ATR-FTIR spectra of compounds eluted from different zones (black lines) and reference standards (dashed lines). (**a**) *Theae folium* zone 2 and EGCG; (**b**) *Theae folium* zone 3, L-theanine and piperidinol derivative (4-But-3-en-1-ynyl-1,2,5-trimethylpiperidin-4-ol); (**c**) *Theae folium* zone 4 and L-glutamic acid; (**d**) *Theae folium* zone 5 and caffeine; (**e**) zone 6 and lignin, hydrolytic; (**f**) *Theae folium* zone 7 and organosolv, lignin, propionate; (**g**) *Theae folium* zone 8, *Theae folium* zone 9 and a long-chain fatty acid ester (stearic acid, ethyl ester).

According to the results, it is evident that the green tea extract provided the highest number of active zones. To characterize bioactive compounds, detected zones of green tea extract were eluted from the preparative TLC chromatogram and further analysed by ATR-FTIR spectrophotometry. The similarity of obtained spectra from Zone 2, with mild COX-1 inhibition, from with the spectrum of epigallocatechin-3-gallate (EGCG) derivative (Figure 3a) [27] suggested the presence of catechins. Literature sources indicate that green tea catechins, particularly galloyl derivatives, are responsible for the inhibitory activity against cyclooxygenases [28]. However, zones at *R*_F_ ≈ 0.2 and 0.4 (Zones 3 and 4), with the highest COX-1 inhibition, show similar FTIR spectra to amino acids, L-theanine and L-glutamic acid, respectively (Figure 2b,d). From a chemical standpoint, green tea contains an approximately 15–20% protein content, encompassing amino acids like L-theanine, tyrosine, tryptophan, threonine, *N*(5)-ethylglutamine, L-glutamic acid, serine, glycine, valine, leucine, aspartic acid, lysine, and arginine [29]. Multiple mechanisms of anti-inflammatory activity of plant polypeptides and amino acids have been described in the literature. Some studies have proposed that bioactive peptides derived from plants, with molecular weights of around 500 Da and composed of two to six amino acids, may possess strong anti-inflammatory properties [30]. Thus, although earlier studies have mostly related green tea’s anti-inflammatory properties to the antioxidant activity of catechins, particularly EGCG, the similarities of the FTIR spectrum of compound from Zone 3 with the highest COX-1 inhibition to the FTIR spectrum of L-theanine and FTIR spectrum of Zone 4 to the FTIR spectrum of glutamic acid, highlight the relevance of amino acids. L-theanine, the main amino acid found in tea leaves, is a non-proteinogenic amino acid. Proteinogenic amino acids and their derivatives make an important group of drug candidates as potential structural analogues of intermediates in metabolism [31]. It has been reported that L-theanine and cystine oral supplements inhibit excessive inflammatory response by lowering the expression of inflammatory proteins and signalling pathways related to inflammation [32]. The spectrum observed in Zone 3 can also be associated with nitrogen-containing heterocyclic compounds (Figure 3b). These heterocyclic compounds with a pyrrole and pyrazine core structures are formed by pyrolysis of amino acids (proline, hydroxy proline, glutamine and asparagine) in the drying stage during the manufacturing of tea [33]. Recent studies have reported that pyrrole and piperazinyl derivatives contain pharmacophore fragments for significant dual COX-1/COX-2 inhibition [34]. Since Zone 4 also exhibits pronounced activity against *E. coli*, we could assume that it originates from the presence of amino acids/peptides. Distinct antimicrobial properties of peptides have been reported. For example, peptides with lysine, arginine or tryptophan in their sequence can disrupt bacterial membranes and inhibit microbial growth [35].

Furthermore, the spectrum of the compound at *R*_F_ ≈ 0.5 closely mirrors the ATR-FTIR spectrum of caffeine (Figure 3d). After catechins and other phenolic compounds, caffeine is one of the most abundant bioactive components in green tea in terms of anticancer and anti-inflammatory properties [36]. In the DPPH• chromatogram of the green tea extract, according to ATR-FTIR spectra, intense zones around *R*_F_ ≈ 0.65–0.80 correspond to lignins (Figure 3e,f). Derivatization of these compounds offered bright red zones for VSA and blue and dark blue zones for NP-PEG. Based on previous work, this class of compounds has been proven to possess significant antioxidant and antimicrobial properties [37,38]. In addition, a slightly weaker inhibitory activity towards COX-1 is noticeable. The ATR-FTIR spectrum confirms that the pronounced antibacterial zones against both tested strains, which are located at the front of the mobile phase, are lipophilic compounds. Specifically in this case, the ATR-FTIR spectrum points to the ethyl ester of stearic acid (Figure 3g). In general, lipophilic compounds can exhibit antimicrobial activity against various microorganisms. This property is a consequence of their ability to disrupt microbial cell membranes, interfere with metabolic processes in the cell, and act as inhibitors of enzymatic activity [39].

## 3. Materials and Methods

### 3.1. Chemicals and Materials

Methanol, ethanol, acetic acid, sulfuric acid, sodium dihydrogen phosphate, sodium chloride, sodium carbonate, Folin-Ciocalteu (FC) reagent, polyethylene glycol 4000 (PEG 4000) and glass HPTLC plates silica gel 60 (Art. 105461) were purchased from Merck (Darmstadt, Germany). Formic acid, ethyl acetate, toluene, Triton X-100, streptomycin, 3-(4,5-dimethylthiazol-2-yl)-2,5-diphenyltetrazolium bromide (MTT), 2,2-di(4-tert-octylphenyl)-1-picrylhydrazyl (DPPH) free radical, vanillin, p-anisaldehyde and 2-aminoethyl diphenylborinate (NP reagent) were from Sigma Aldrich Chemie GmbH (Steinheim, Germany). Standards of rutin, gallic acid and Trolox were supplied by Sigma Aldrich Chemical Co. (St. Louis, MO, USA). Nutrient agar slants were provided from Lab M (Bury, UK), and Tripton LP0042 and yeast extract LP0021 was obtained from Oxoid LTD (Basingstoke, UK). Supelco preparative TLC silica gel 60 F254, 20 × 20 cm, glass plates, 1.5 mm thick, were purchased from Merck Pty. Ltd. (Bayswater, Australia). Arachidonic acid, cyclooxygenase-1 (COX-1) from sheep (1500 U/mg), dimethyl sulfoxide (DMSO) (≥99.7%), hemin, salicylic acid and Wurster’s blue (*N*,*N*,*N*′,*N*′-tetramethyl-p-phenylenediamine) were obtained from Sigma-Aldrich (Macquarie Park, Australia).

### 3.2. Plant Material and Extraction of Secondary Metabolites

Nineteen samples of medicinal plants were obtained as commercially available medicinal herbal teas, collected, and sold by the Institute for Medicinal Plant Research “Dr. Josif Pančić”, Belgrade, Serbia (Table 2). For each extract, 10 g of finely ground plant material was suspended in 100 mL of absolute ethanol in a 250 mL Erlenmeyer flask, placed into an ultrasonic bath at room temperature and extracted for 30 min. The obtained extracts were filtered and evaporated on a rotary evaporator. The resulting residue was dissolved in sufficient methanol to produce an extract concentration of 50 mg/mL.

### 3.3. Planar Chromatography

Plant extracts were applied as 6 mm bands onto 20 cm × 10 cm HPTLC glass silica gel plates using a Linomat 5 (CAMAG, Muttenz, Switzerland). The bands were applied starting from the lower edge (8 mm) and with a minimum distance of 13 mm from each side. Plates were developed to a distance of 80 mm in a saturated Twin Trough Chamber 20 cm × 10 cm, with a mobile phase composed of ethyl acetate: toluene: formic acid: water (16:4:3:2, *v*/*v*/*v*/*v*). The digital images of chromatograms were recorded with a TLC Visualizer documentation system (CAMAG) operated with winCATS software (version 1.4.4.6337, CAMAG) and evaluated for quantification with VideoScan (version 1.02, CAMAG).

For preparative TLC, a single band of about 50 mg of dried extract was applied on the plate after being dissolved in a small volume of solvent. After plate development, nine detected zones (Zone 1, *R*_F_ = 0.04–0.09; Zone 2, *R*_F_ = 0.10–0.16; Zone 3, *R*_F_ = 0.16–0.27; Zone 4, *R*_F_ = 0.33–0.44; Zone 5, *R*_F_ = 0.44–0.55; Zone 6, *R*_F_ = 0.67–0.76; Zone 7, *R*_F_ = 0.76–0.82; Zone 8, *R*_F_ = 0.87–0.94 and Zone 9, *R*_F_ = 0.94–1.0) were scraped from the plate and eluted with ethyl acetate through a coarse fritted funnel. The recovered eluate was then allowed to stand overnight at room temperature to concentrate by evaporation.

### 3.4. Chemical Derivatization

Vanillin–sulfuric acid (VSA) reagent was obtained by dissolving 250 mg of vanillin in 90 mL of absolute ethanol, with the subsequent addition of 10 mL of sulfuric acid. The developed HPTLC chromatograms were sprayed with a prepared solution by using a TLC sprayer, heated to 105 °C for 5 min until bands were visualized, and then evaluated under white light. 

A natural product (NP) reagent was produced by dissolving 500 mg of 2-aminoethyl diphenylborinate in 100 mL of methanol. After development, the HPTLC chromatograms were subjected to a sequential spraying process: first with the NP solution, followed by drying with a stream of hot air, and then with a 5% (*w*/*v*) polyethylene glycol 4000 (PEG) solution in methanol. Plates were evaluated under white light. 

A 0.25% (*w*/*v*) solution of DPPH• was prepared in methanol and used for spraying the developed plate. After a 30 min incubation at room temperature in darkness, the plate was examined under white light.

A neutral ferric chloride solution was obtained by a dropwise addition of dilute solution of sodium hydroxide to a 2% (*w*/*v*) solution of ferric chloride in methanol until a reddish brown precipitate of ferric hydroxide started to precipitate. The resulting precipitate was separated by filtration, and the solution was used for derivatization. After the reagent was sprayed on the layer, the plate was heated for 2–3 min at 110 °C and recorded under white light.

Aluminium chloride solution was prepared by dissolving 2 g of AlCl_3_ in 100 mL of methanol. After derivatization, flavonoids were seen as bright blue, fluorescent zones under UV 360 nm.

### 3.5. COX-1 Inhibitory Assay

A COX-1 enzyme stock solution was prepared in DMSO. Enzyme assay working solution was then prepared by diluting the enzyme stock solution with 100 mmol/L Tris buffer (pH 7.4) and adding hemin as a cofactor to make a final solution containing 1 unit/mL of COX-1 and 1 µmol/L of hemin. The reaction mixture solution was produced by diluting an arachidonic acid stock solution (1 mmol/L in ethanol) with the buffer and adding Wurster’s blue stock solution (2 mmol/L in DMSO) to obtain the final mixture of 5 µmol/L arachidonic acid and 1 mmol/L of Wurster’s blue. Using a TLC reagent sprayer, the developed plate was sprayed with the enzyme solution and then incubated at 37 °C in a humidity chamber for 20 min. Color reaction was initiated after spraying the plate with the reaction mixture solution. After 5–10 min, zones of COX-1 inhibition were detected as white bands on a violet background. Salicylic acid was used as a standard reference to evaluate COX-1 inhibition.

### 3.6. Antibacterial Assays

Luria Bertani (LB) broth was prepared by adding 10 g of tryptone, 5 g of yeast extract and 5 g of sodium chloride to 1 L of distilled water and autoclaving it at 121 °C. 

HPTLC bioautography assays were carried out against *Escherichia coli* ATCC 35,218 and *Staphylococcus aureus* ATCC 6538. Standard bacterial strains were cultivated on nutrient agar slants for 24 h at 37 °C. Each well-grown culture was suspended in 5 mL of sterile physiological solution. A 10 mL LB broth was inoculated with 0.1 mL of the obtained bacterial cell suspension and incubated overnight (18 h) on a BioSan Orbital Shaker-Incubator ES-20 at 37 °C and 220 rpm. The bacterial suspensions for derivatization were prepared by inoculating 200 mL of LB in 500 mL flasks with 0.2 mL bacterial cultures grown overnight. The flasks were incubated in the incubator shaker at 37 °C until the suspensions reached the exponential phase of growth (OD = 0.6). The developed plates were immersed in bacterial suspensions (OD = 0.6) for a few seconds and then incubated in a humidity chamber at 37 °C under aerobic conditions for 100 min, allowing the bacteria growth on the plate surface. Antibacterial zones were visualized using a thermostatted 0.1% MTT solution in a phosphate buffer (0.1 mol/L, pH 7.2). In the case of the *E. coli* test, 0.1 mL of Triton X-100 was added to the MTT solution. An additional 60 min incubation was performed, and positive reactions, indicated by a color change, were noted. Streptomycin was used as a reference standard for these assays.

### 3.7. ATR-FTIR Spectroscopy

The absorbance spectra of eluted compounds from the zones of interest were measured with a Cary 630 FTIR equipped with a single reflection Diamond ATR sampling accessory (Agilent Technologies Pty Ltd., Mulgrave, Australia). The spectrum was collected in the region between 4000 and 650 cm^−1^ at a resolution of 4 cm^−1^ and 64 scans per sample. To measure the spectrum, a few drops of concentrated eluate from the sample zone were placed onto the ATR crystal, and once the solvent evaporated, the FTIR spectrum was recorded. The spectrum of each sample was corrected against a background spectrum of air. Compounds from the eluted zones were characterized based on spectral library searching against the in-house collection at La Trobe University.

### 3.8. Spectrophotometric Assays

For the spectrophotometric assays, a UV–Visible Cintra 6 spectrophotometer (GBC Scientific Instruments, Keysborough, Australia) was used. The total phenolic content (TPC) in the extracts was determined with the Folin–Ciocalteu method and with gallic acid, in the concentration range of 10–120 mg/L, as the reference standard. For each sample, 0.5 mL of sample extract was added to 0.5 mL of ultrapure water, and the solution was mixed with 2.5 mL of a 10% *v*/*v* Folin-Ciocalteu reagent. After incubating for 5 min, 2.0 mL of a 7.5% *w*/*w* sodium carbonate solution was added. After a 2 h incubation, the absorbance was measured at 765 nm. TPC was expressed as milligrams of gallic acid equivalent (GAE) per gram of dry extract. 

The total flavonoid content (TFC) in the extracts was determined with the aluminium chloride method and rutin as the reference standard in the concentration range of 10–100 mg/L. In a 10 mL test tube, 0.3 mL of sample extract, 3.4 mL of 30% (*v*/*v*) methanol, 0.15 mL of 0.5 mol/L sodium nitrite, and 0.15 mL of 0.3 mol/L AlCl_3_ were mixed. After 5 min, 1.0 mL of a 1 mol/L sodium hydroxide solution was added and the absorbance was measured at 506 nm. TFC was expressed as milligrams of rutin equivalent (RUE) per gram of dry extract. 

Radical scavenging activity (RSA) was assessed using the DPPH• solution. A volume of 0.1 mL of sample extract was mixed with 4 mL of a 71 μmol/L DPPH• in methanol. The obtained solution was incubated for 45 min at room temperature, and its absorbance was measured at 517 nm. Trolox (6-hydroxy-2,5,7,8-tetramethylchroman-2-carboxylic acid) was used as an antioxidant standard (0.025–0.15 mg/L). The *RSA* (%) was expressed as a percentage of DPPH• discoloration using the following equation:

(1) $$RSA\;(\%)=\frac{A_{DPPH}-A_{sample}}{A_{DPPH}}\times 100,$$

where *A_DPPH_* is absorbance of the DPPH• solution in methanol and *A_sample_* is sample absorbance. The obtained results were presented as milligrams of Trolox equivalent (TE) per gram of dry extract.

### 3.9. Method Validation

The methods for determination of gallic acid, rutin, streptomycin, salicylic acid, and Trolox were validated as per the guidelines established by the International Conference on Harmonization (ICH). For the HPTLC assays, the working range was determined by plotting chromatographic peak areas against standard concentrations (expressed in μg/band). In the case of colorimetric assays, the working range was established by plotting the standard concentration (mg/L) against the corresponding absorbance. Linear ranges were determined using least square regression analysis. Precision was expressed as relative standard deviation (%RSD) of three replicates at three concentration levels within the calibration curve. Sensitivity of the method was evaluated with the limit of quantification (LOQ) and limit of detection (LOD) that were calculated based on the standard deviation of the response and the slope of the calibration line.

## 4. Conclusions

Our results justify the historical use of investigated plants in the treatment of anti-inflammatory and infectious diseases. The results confirmed that the anti-inflammatory activity is associated with the presence of flavonoids, while antimicrobial activity is associated with the presence of fatty acids. However, the superior anti-inflammatory activity of green tea leaf extracts is attributed to amino acids and heterocyclic compounds with pyrrole and pyrazine core structures. Many heterocyclic compounds, mostly pyrrole and pyrazine derivatives, are formed during the drying processing of green tea leaves. They are an important component that contributes to the formation of tea aroma quality. The pyrrole moiety is an established scaffold for mixed COX-1/COX-2 inhibitors that is more effective in reducing signs of inflammation than a selective inhibition of COX-2, and with less side effects [78].

In addition, the proposed approach based on HPTLC chemical derivatization provided a phytochemical comparison of plant extracts, while hyphenation with bioassays enabled screening for and detection of the bioactive compounds. Further coupling with ATR-FTIR enabled the structural characterization of the compound with the strongest activity.

## Figures and Tables

**Table 1:** Antioxidant, antibacterial activity, COX-1 inhibition, total phenolic and flavonoid content expressed as milligrams of standards per gram of dry extract.

Sample	TPC	TFC	RSA	COX-1	*S. aureus*	*E. coli*	DPPH•	FeCl_3_	AlCl_3_
	GAE (mg/g)	RUE (mg/g)	TE (mg/g)	SAE (mg/g)	StrpE (mg/g)	StrpE (mg/g)	GAE (mg/g)	GAE (mg/g)	RUE (mg/g)
1	57.7	24.8	98.7	21,363.0	470.4	2146.2	161.4	196.6	132.6
2	237.9	98.3	571.1	74,121.4	1594.8	5620.6	935.7	695.2	219.3
3	37.3	32.4	50.5	37,332.0	1405.0	1736.5	170.4	243.2	208.5
4	41.6	87.0	63.5	40,756.1	1543.6	2680.2	202.6	237.6	180.2
5	38.1	104.5	65.3	51,571.7	885.2	3751.8	169.0	461.5	239.2
6	19.9	92.3	18.7	28,924.8	3555.5	5443.9	203.9	365.0	162.8
7	17.0	40.9	10.9	47,790.6	1822.0	1587.6	7.0	442.9	18.8
8	132.8	161.5	214.3	11,470.7	539.3	2177.9	815.8	730.3	596.2
9	134.0	130.0	248.3	13,807.7	815.2	1308.1	706.2	167.8	39.3
10	17.8	32.5	13.7	30,069.2	1417.2	1601.1	16.0	165.1	271.2
11	81.6	40.1	127.6	58,022.4	998.6	3872.7	688.5	535.5	472.2
12	72.3	35.2	135.1	50,226.1	2191.9	3787.5	529.3	416.9	328.8
13	53.7	17.9	59.9	22,730.5	2001.3	1434.3	417.0	255.7	278.3
14	30.4	26.3	22.5	50,989.9	2850.3	2617.3	91.1	148.4	177.2
15	35.5	10.1	33.1	8055.9	1417.1	1459.6	127.1	371.5	512.5
16	24.3	30.8	35.6	27,849.1	1986.7	2703.3	90.0	346.2	74.1
17	47.1	52.0	42.2	29,343.9	1895.7	1920.4	188.5	170.2	160.4
18	140.8	109.3	155.9	16,577.4	667.3	606.0	678.2	719.7	162.9
19	16.7	31.1	18.5	22,584.6	1222.7	858.6	22.2	253.0	269.3

**Table 2:** Investigated medicinal plants and their anti-inflammatory and antimicrobial potential according to the literature.

	Sample, Plant Species and Family	Anti-Inflammatory Potential	Antimicrobial Potential
1.	Rosemary leaf, *Rosmarinus officinalis* L. (Lamiaceae)	[40]	[41]
2.	Green tea leaf, *Camellia sinensis* L. (Theaceae)	[42]	[43]
3.	Elder flower, *Sambucus nigra* L. (Caprifoliaceae)	[44]	[45]
4.	Plantain leaf, *Plantago major* L. (Plantaginaceae)	[46]	[47]
5.	Sage leaf, *Salvia officinalis* L. (Lamiaceae)	[48]	[49]
6.	Nettle leaf, *Urtica dioica* L. (Urticaceae)	[50]	[51]
7.	Calendula flower, *Calendula officinalis* L. (Asteraceae)	[52]	[53]
8.	Walnut leaf, *Juglans regia* L. (Juglandaceae)	[54]	[55]
9.	St. John’s wort herb, *Hypericum perforatum* L. (Hypericaceae)	[56]	[57]
10.	Mallow flower, *Malva silvestris* L. (Malvaceae)	[58]	[59]
11.	Wild thyme herb, *Thymus serpyllum* L. (Lamiaceae)	[60]	[61]
12.	European Goldenrod herb, *Solidago virgaurea* L. (Asteraceae)	[62]	[63]
13.	Chamomile flower, *Matricaria chamomilla* L. (Asteraceae)	[64]	[65]
14.	Greater celandine herb, *Chelidonim majus* L. (Papaveraceae)	[66]	[67]
15.	Fumitory herb, *Fumaria officinalis* L. (Fumariaceae)	[68]	[69]
16.	Comfrey root, *Symphytum officinale* L. (Boraginaceae)	[70]	[71]
17.	Cowslip flower, *Primula veris* L. (Primulaceae)	[72]	[73]
18.	Immortelle flower, *Helichrysum arenarium* L. (Asteraceae)	[74]	[75]
19.	Dandelion leaf, *Taraxacum officinale* L. (Asteraceae)	[76]	[77]

## Data Availability

The data presented in this study are available on request from the corresponding authors.

## Supplementary Materials

The following supporting information can be downloaded at: https://www.mdpi.com/article/10.3390/molecules28217346/s1, Table S1: Regression data for HPTLC assays; title; Table S2: Method precision in terms of relative standard deviations (RSD) for repeated measurements (*n* = 3); Table S3: Regression data for colorimetric assays.
