# Supplementary material for: Bioassay-Guided Assessment of Antioxidative, Anti-Inflammatory and Antimicrobial Activities of Extracts from Medicinal Plants via High-Performance Thin-Layer Chromatography
Source: Molecules. 2023 Oct 30;28(21):7346. doi: 10.3390/molecules28217346 (PMC10647317; doi:10.3390/molecules28217346)
Supplement: Supplementary file 1 [file molecules-28-07346-s001.zip › molecules-2631025-supplementary.pdf]

Supplementary material

Bioassay-Guided Assessment of Antioxidative, Anti-Inflammatory and Antimicrobial Activities of Extracts from Medicinal Plants via High Performance Thin Layer Chromatography

Marko D. Jović [1], Snezana Agatonovic-Kustrin [2,3,*], Petar M. Ristivojević [4,*], Jelena Đ. Trifković [4] and David W. Morton [2,3]

[1] Innovation Centre of the Faculty of Chemistry Ltd., University of Belgrade, Studentski Trg 12-16, 11158 Belgrade, Serbia

[2] Department of Pharmaceutical and Toxicological Chemistry named after Arzamastsev of the Institute of Pharmacy, I.M. Sechenov First Moscow State Medical University (Sechenov University), 119991 Moscow, Russia

[3] Department of Rural Clinical Sciences, La Trobe University, Edwards Road, Bendigo 3550, Australia

[4] Department of Analytical Chemistry, Centre of Excellence for Molecular Food Sciences, University of Belgrade, Studentski trg 12-16, 11158 Belgrade, Serbia

Table S1. Regression data for HPTLC assays.

Method	Standard	Regression equation	R^2	Linear range (μg)	LOD (μg)	LOQ (μg)
COX-1	Salicylic acid	y=53.269x + 33277	0.982	20-5000	519.0	1572.8
S. aureus	Streptomycin	y=8722.3x + 80905	0.987	2.5-50	6.5	19.7
E. coli	Streptomycin	y=10779x - 19261	0.989	2.5-45	5.2	15.9
DPPH	Gallic acid	y=11388x + 45743	0.988	0.5-6	0.9	2.6
$FeCl_3$	Gallic acid	y=11854x + 26440	0.975	1-10	1.8	5.3
$AlCl_3$	Rutin	y=8407.9x + 38043	0.974	1-7	1.3	3.8

Table S2. Method precision in terms of relative standard deviations (RSD) for repeated measurements (*n*=3).

Spectrophotometry						HPTLC									
TPC (Gallic acid)		TFC (Rutin)		RSA (Trolox)		COX-1 (Salicylic acid)		*S. aureus* (Streptomycin)		*E. coli* (Streptomycin)		DPPH (Gallic acid)		$FeCl_3$ (Gallic acid)		$AlCl_3$ (Rutin)	
c (mg/mL)	RSD (%)	c (mg/mL)	RSD (%)	c (mg/mL)	RSD (%)	Applied (μg)	RSD (%)	Applied (μg)	RSD (%)	Applied (μg)	RSD (%)	Applied (μg)	RSD (%)	Applied (μg)	RSD (%)	Applied (μg)	RSD (%)
15.0	2.85	20.0	9.16	100.0	10.68	400.0	10.10	5.0	13.86	5.0	15.70	0.5	3.63	1.0	4.44	1.0	4.77
60.0	1.77	50.0	6.71	300.0	6.64	1000.0	6.69	25.0	11.54	25.0	10.24	3.0	4.47	5.0	1.23	6.0	3.03
125.0	1.41	100.0	4.71	500.0	6.65	2000.0	3.88	50.0	10.52	45.0	11.56	5.0	2.38	7.0	1.06	7.0	2.07
Average	2.01		6.86		7.99		6.89		11.97		12.50		3.49		2.24		3.29

Table S3. Regression data for colorimetric assays.

Method	Standard	Regression equation	R^2	Linear range (mg/L)	LOD (mg/L)	LOQ (mg/L)
TPC	Gallic acid	y=0.0085x + 0.0408	0.998	10-250	14.2	43.1
TFC	Rutin	y=0.00065x + 0.00327	0.985	10-100	12.1	36.8
RSA	Trolox	y=267.3x-0,5509	0.993	0.025-0.15	0.015	0.045
